# Oligonol Supplementation Affects Leukocyte and Immune Cell Counts after Heat Loading in Humans

**DOI:** 10.3390/nu6062466

**Published:** 2014-06-24

**Authors:** Jeong Beom Lee, Young Oh Shin

**Affiliations:** 1 Department of Physiology, College of Medicine, Soonchunhyang University, 366-1, Ssangyong-dong, Cheonan, 331-946, Korea; E-Mail: leejb@sch.ac.kr; 2 Department of Health Care, Global Graduate School, Soonchunhyang University, 646, Eupnae-ri, Shinchang-myun, Asan-si, Chungnam 336-745, Korea

**Keywords:** oligonol, leukocyte, lymphocyte, immune cell, heat

## Abstract

Oligonol is a low-molecular-weight form of polyphenol and has antioxidant and anti-inflammatory activity, making it a potential promoter of immunity. This study investigates the effects of oligonol supplementation on leukocyte and immune cell counts after heat loading in 19 healthy male volunteers. The participants took a daily dose of 200 mg oligonol or a placebo for 1 week. After a 2-week washout period, the subjects were switched to the other study arm. After each supplement, half-body immersion into hot water was made, and blood was collected. Then, complete and differential blood counts were performed. Flow cytometry was used to enumerate and phenotype lymphocyte subsets. Serum concentrations of interleukin (IL)-1β and IL-6 in blood samples were analyzed. Lymphocyte subpopulation variables included counts of total T cells, B cells, and natural killer (NK) cells. Oligonol intake attenuated elevations in IL-1β (an 11.1-fold change *vs.* a 13.9-fold change immediately after heating; a 12.0-fold change *vs.* a 12.6-fold change 1h after heating) and IL-6 (an 8.6-fold change *vs.* a 9.9-fold change immediately after heating; a 9.1-fold change *vs.* a 10.5-fold change 1h after heating) immediately and 1 h after heating in comparison to those in the placebo group. Oligonol supplementation led to significantly higher numbers of leukocytes (a 30.0% change *vs.* a 21.5% change immediately after heating; a 13.5% change *vs.* a 3.5% change 1h after heating) and lymphocytes (a 47.3% change *vs.* a 39.3% change immediately after heating; a 19.08% change *vs.* a 2.1% change 1h after heating) relative to those in the placebo group. Oligonol intake led to larger increases in T cells, B cells, and NK cells at rest (*p* < 0.05, *p* < 0.05, and *p* < 0.001, respectively) and immediately after heating (*p* < 0.001) in comparison to those in the placebo group. In addition, levels of T cells (*p* < 0.001) and B cells (*p* < 0.001) were significantly higher 1 h after heating in comparison to those in the placebo group. These results demonstrate that supplementation with oligonol for 1 week may enhance the immune function under heat and suggest a potential useful adjunct to chemotherapy in malignant diseases.

## 1. Introduction

The immune system performs a protective function against external pathogens or stressors. Several studies have demonstrated that the adequate intake of vitamins and antioxidants is crucial for the efficient functioning of the immune system [1,2,3]. In this regard, the present study hypothesizes that the antioxidant oligonol affects the human immune system.

Oligonol is a novel compound produced from the oligomerization of polyphenol and is an optimized phenolic product containing catechin-type monomers and oligomers (dimers, trimers, and tetramers) of proanthocyanidin that are easily absorbed [4]. In the last decade, several studies have demonstrated the antioxidant and anti-inflammatory activity of oligonol [5,6,7,8]. However, the effects of oligonol supplementation on immunity remain unclear. A study of effects of the long-term supplementation of oligonol (100 mg per day for a month) on the resting level of leukocytes found no significant effect [5].

The present study considers an experimental protocol consisting of 1 week of oligonol supplementation (200 mg per day) and acute heat stress, including half-body immersion in hot water, because heat stress stimulates the immune function and induces some disturbance, including leukocytosis, granulocytosis, monocytosis, and lymphocytosis [9].

Passive hyperthermia (a rise in the body’s core temperature) is a consequence of a heat gain in excess of the body’s ability to dissipate it and is observed under environmental heat loading such as hot-water immersion. Hyperthermia results in the significant activation of immune cells and cytokine production. Circulating pro-inflammatory cytokines, including interleukin (IL)-1β and IL-6, are “endogenous pyrogens” involved in the febrile response during infection or inflammation [10]. Therefore, it is necessary to test the anti-inflammatory effect of oligonol in response to heat stress.

The present study evaluates the effects of oligonol supplementation on immune modulation in human subjects undergoing some heat load by determining leukocyte and immune cell counts as well as serum concentrations of IL-1β and IL-6.

## 2. Experimental Section

A placebo-controlled cross-over design was employed. The subjects consumed oligonol (200 mg per day) or a placebo for 1 week. Two periods were separated by a 2-week washout. Half-body immersion in hot water was conducted as a heat load at the end of each period.

### 2.1. Subjects

This experimental protocol was approved by the University of Soonchunhyang Research Committee and a written informed consent form was obtained from all participants after the purpose of the study, experimental procedures, and any potential risks were thoroughly discussed. A total of 19 healthy male college students (age: 23.7 ± 2.3 years; height: 174.5 ± 4.1 cm; weight: 68.9 ± 5.2 kg; body mass index: 19.8 ± 3.0 kg/m^3^; body fat: 16.4% ± 3.2%; VO_2max_: 52.1 ± 4.3 mL/kg/min) were enrolled. The protocol was in compliance with the 1975 Declaration of Helsinki. The subjects refrained from alcohol consumption, smoking, medication, and vigorous physical activity during the testing period.

### 2.2. Supplements

Oligonol is produced by the oligomerization of polyphenols found abundantly in lychees. Typically, the constituents of oligonol include 15%–20% monomers, 8%–12% dimers, and 5%–10% trimers. In this study, oligonol was supplied by Amino Up Chemical Company (Sapporo, Japan). The dose of oligonol (200 mg) was determined previously to be safe for the repeated intake of doses <200 mg/day [11]. Starch was used as a placebo.

### 2.3. Heat Load

All experiments were conducted in a thermoneutral climate chamber (26 ± 0.5 °C; 60% ± 3% relative humidity; air velocity < 1 m/s) from 2 to 5 p.m. Upon their arrival at the climate chamber, the subjects wore shorts and sat in a chair in a relaxed posture for 60 min to become conditioned to the chamber climate prior to the commencement of the experiment. After this rest period, a heat load was applied to each subject through the immersion of half of the body in a hot water bath maintained at 42 ± 0.5 °C for 30 min. Blood was collected at rest, immediately after immersion and 1 h after immersion. Because the water temperature was uncomfortably hot, the subjects were allowed to take breaks as short as 1min at 5, 10, and 20 min checkpoints during the 30 min immersion. The subjects consumed no caffeine, tobacco, or alcohol 48 h before the test and refrained from any intense physical activity 24 h before the test.

### 2.4. Blood Sampling

Blood samples were collected from the antecubital vein of the subjects according to the guidelines of the Clinical and Laboratory Standards Institute to identify changes in serum markers 1 h prior to, immediately after, and 1 h after immersion. Blood was transferred to SST tubes and immediately centrifuged at 4 °C and 3000 rpm for 10 min. The serum was subsequently removed and stored in 1 mL aliquots at −80 °C until its analysis.

### 2.5. Analysis of Serum IL-1ß and IL-6

IL-1β and IL-6 were measured in duplicates by an enzyme-linked immunosorbent assay (R & D Systems, Minneapolis, MN, USA). The intra-assay coefficient of variation (CV) ranged from 3.2% to 10.0%, and the inter-assay CV, from 4.5% to 9.0%. Values below this limit were assumed to be zero for the statistical analysis.

### 2.6. Full Blood Counts

Full blood counts were performed using an AcT 5 diff AL five-part differential hematology analyzer (Beckman Coulter, Palo Alto, CA, USA). This hematology analyzer uses a sequential dilution system and a dual focused flow fluid dynamic technology employing the Coulter principle of impedance to count and size cells.

### 2.7. Assessment of Lymphocyte Subsets

Lymphocyte subsets were assessed using the IOTest kit (Beckman Coulter, Miami, FL, USA). The IOTest kit quantitatively and qualitatively measured the number of blood lymphocytes. This kit included reagents containing phycoerythrin-conjugated monoclonal antibodies specific for T (CD3), B (CD19), and natural killer (NK) (CD16/CD56) lymphocytes. Samples were analyzed according to the manufacturer’s instructions.

### 2.8. Tympanic Temperature Measurement

Tympanic temperature (Tty) was assessed in the left ear through the insertion of a thermistor probe (TSK7 + 1, Songkitopia, Incheon, Korea) with a small spring into the ear canal (TAKARA, instrument Co. Ltd., Yokohama, Japan). Here the probe was connected to a personal computer (model CF-T1, Panasonic, Tokyo, Japan) as well as to the model K-720 data logger (Technol Seven, Yokohama, Japan).

### 2.9. Statistical Analysis

Descriptive statistics are expressed as the mean ± standard deviation based on SPSS for Windows ver. 12.0 (SPSS Inc., Chicago, IL, USA). A repeated two-way analysis of variance was conducted to compare values, and a contrast method was employed to compare values within each group. The level of significance was set to *p* < 0.05.

## 3. Results

### 3.1. Serum Concentrations of IL-1ß and IL-6

**Figure 1:**
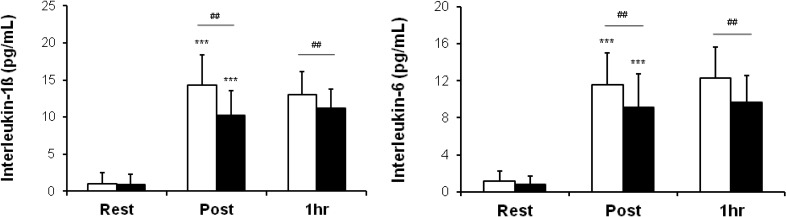
Serum concentrations of interleukin (IL)-1β and IL-6 over time in two groups. Rest: before heating; Post: immediately after heating; 1 h: 1 h after heating. White columns indicate placebo intake, and black columns, oligonol intake. Values are presented as the mean ± standard deviation. *** *p* < 0.001 indicates a significant difference in comparison to Rest within the same group, and **^##^** *p* < 0.01 indicates a significant difference between two groups.

Figure 1 shows the mean serum concentrations of IL-1β and IL-6 over time in the two groups. There were significant increases in all values immediately after heating in both groups. However, oligonol intake attenuated elevations in IL-1β (an 11.1-fold change *vs.* a 13.9-fold change immediately after heating; a 12.0-fold change *vs.* a 12.6-fold change 1h after heating) and IL-6 (an 8.6-fold change *vs.* a 9.9-fold change immediately after heating; a 9.1-fold change *vs.* a 10.5-fold change 1 h after heating) immediately and 1 h after heating in comparison to the placebo group.

### 3.2. Leukocytes, Lymphocytes, and Cell Subsets

**Figure 2:**
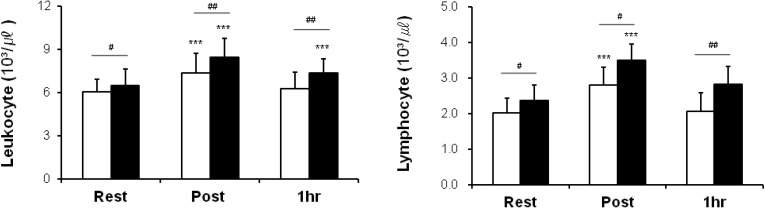
Leukocytes and lymphocytes over time in two groups. Rest: before heating; Post: immediately after heating; 1 h: 1 h after heating. White columns indicate placebo intake, and black columns, oligonol intake. Values are presented as the mean ± standard deviation. *** *p* < 0.001 indicates a significant difference in comparison to Rest within the same group, and **^#^** *p* < 0.05 and **^##^** *p* < 0.01 indicate significant differences between two groups.

**Figure 3:**
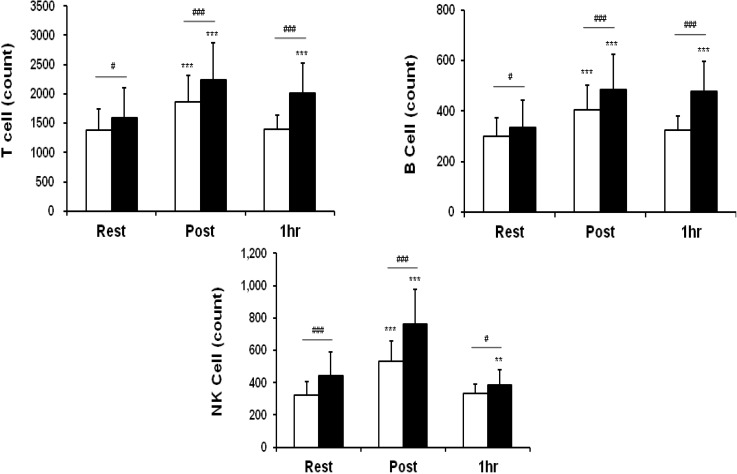
Lymphocyte subset counts over time in two groups. Rest: before heating; Post: immediately after heating; 1 h: 1 h after heating. White columns indicate placebo intake, and black columns, oligonol intake. Values are presented as the mean ± standard deviation. ** *p* < 0.01 and *** *p* < 0.001 indicate significant differences in comparison to Rest within the same group, and **^#^** *p* < 0.05 and **^###^** *p* < 0.001 indicate significant differences between two groups.

Differences between the placebo and oligonol groups are shown in Figure 2 and Figure 3. Mean leukocyte and lymphocyte values of these two groups were significantly different at all checkpoints. Oligonol supplementation led to significant increases in numbers of leukocytes (a 30.0% change *vs.* a 21.5% change immediately after heating; a 13.5% change *vs.* a 3.5% change 1 h after heating) and lymphocytes (a 47.3% change *vs.* a 39.3% change immediately after heating; a 19.08% change *vs.* a 2.1% change 1 h after heating) in comparison to those in the placebo group.

Counts of T, B and NK cells at rest showed a greater acceleration in the oligonol group than in the placebo group. In addition, oligonol intake led to larger increases in T cells (a 41.4% change *vs.* a 34.3% change immediately after heating; a 27.4% change *vs.* a 0.9% change 1h after heating), B cells (a 45.5% change *vs.* a 35.1% change immediately after heating; a 42.8% change *vs.* an 8.1% change 1 h after heating) and NK cells (a 71.7% change *vs.* a 63.9% change immediately after heating; a −13.6% change *vs.* a 3.1% change 1h after heating) immediately and 1 h after heat loading in comparison to those in the placebo group.

### 3.3. Tympanic Temperature

The tympanic temperature of both trials increased significantly from the resting level immediately after the heat load. However, Oligonol intake led to a lower tympanic temperature than the placebo trial (a change from 36.74 ± 0.23 °C to 37.70 ± 0.30 °C *vs.* that from 36.68 ± 0.18 °C to 37.84 ± 0.32 °C, *p* < 0.01).

## 4. Discussion

This study evaluates the effects of oligonol on immunity through the regulation of immune cells in response to heat. According to the results, oligonol supplementation attenuated elevations in tympanic temperature and serum levels of the pro-inflammatory cytokines IL-1β and IL-6 after heating. Total leukocyte and lymphocyte counts showed significant increases at rest and after heat loading in comparison to those in the placebo group. Oligonol intake led to significant increases in T, B, and NK cells at rest and immediately after heat loading, and levels of T and B cells were significantly higher 1 h after immersion in comparison to those in the placebo group. These results suggest that oligonol may be used as a type of adjuvant for improving host immunity. This study is the first to demonstrate immune modulation by oligonol supplementation.

Hyperthermia increases the pro-inflammatory cytokines IL-6 [5,6,12] and IL-1β [5,6,13]. This study’s results are consistent with the findings of studies showing heat-induced incremental increases in IL-6 and IL-1β. It is well documented that pro-inflammatory cytokines such as IL-1β, IL-6, and interferon-γ increase in response to stress [14,15]. IL-1β plays a crucial role in the cytokine network during the immune response [16]. IL-6, a pro- and anti-inflammatory cytokine, plays a crucial role in the host immune response, acute protein synthesis, and the maintenance of homeostasis. In addition, IL-1β and IL-6, pyrogenic cytokines, are involved in febrile and inflammatory responses [5,17]. Consistent with the findings of previous research on the thermally induced expression of IL-1β and IL-6 [5,6], the results of the present study show a similar pattern. However, the result showing that oligonol intake attenuated heat-induced increases in these cytokines provides support for the anti-inflammatory effect of oligonol. In addition, oligonol also had an antipyretic effect. Oligonol intake attenuated incremental increases in tympanic temperature in comparison to the placebo trial, which is consistent with the findings of previous research [18].

Hyperthermia plays an important role in the immune system function [19]. Heat exposure, including hot-water immersion and irradiation, can induce leukocytosis, granulocytosis, minor monocytosis, and lymphocytosis [20,21,22]. Because the circulating numbers and functional capacity of leukocytes may affect the immune response, the present study analyzes leukocyte and individual lymphocyte counts and observes leukocytosis and lymphocytosis after heat loading. In particular, total leukocyte and lymphocyte counts after oligonol supplementation showed larger increases than those in the placebo group. Immune modulation was observed in lymphocyte subsets. Oligonol intake led to significant increases in T, B, and NK cells at rest and immediately after heat loading, and levels of T and B cells were significantly higher 1h after heating than those in the placebo group. These results suggested that oligonol enhanced the immune system, although the underlying mechanism remains unclear. Hyperthermia-induced leukocytosis was associated with significant changes in the absolute number and percentage of circulating lymphocyte subpopulations. T-suppressor cytotoxic and NK cells have been shown to show significant increases in heatstroke patients [20]. In addition, the activation of NK and T cells by hyperthermia has been observed [23,24,25]. The present study’s results show a similar pattern of a lymphocyte subset distribution found in previous research [20,23,24,25].

T lymphocytes play a central role in mounting and regulating the response to both intra- and extra-cellular pathogens. Several studies have found that hyperthermia induces T cells activation [20,23]. In the present study, the number of T cells increased after heating in both trials, and this was facilitated by oligonol. B cells are known mainly for playing a pivotal role in the production of antibodies against a broad range of antigens. The sign of B-cell activation can be determined by the induction of immunoglobulin production. Although immunoglobulin production was not measured in this study, the number of B cells increased after heating in both trials, and this was facilitated by oligonol. The results for B cells are consistent with the findings of Huang *et al.* [26], who demonstrated that the significant activation of B cells by hyperthermia. NK cells are thought to play an important role as the first line of defense against infectious and malignant diseases. Hyperthermia in humans and animals has been shown to increase NK-cell activity as well as the total number of NK cells [27,28]. The present study’s results for NK cells are consistent with the findings of these studies. In particular, oligonol intake helped to enhance the modulation of lymphocyte subsets.

Fever (hyperthermia) is a key element in the acute-phase response and is generally beneficial against bacterial, fungal, and viral infection (e.g., sepsis), even in the case of lipopolysaccharide-induced fever [29,30]. Previous studies of induced hyperthermia in infected animals have suggested that an increase in the body’s core temperature enhances immunity. However, the underlying mechanism through which an increase in the core temperature improves host survival remains unclear. Nevertheless, it is generally accepted that the expression of cytokines plays an important role in the disease process. IL-1β and tumor necrosis factor alpha (TNF-α) are predominantly pro-inflammatory, and IL-6 is a pleiotropic cytokine produced by a wide range of cell types, including leukocytes. According to previous research [29], fever enhances the specific tissue expression of IL-1β and TNF-α, whereas it enhances the generation of IL-6 in most tissue types. IL-6 plays an important role in limiting local and systemic inflammation by producing negative feedback, which results in an anti-inflammatory and immunosuppressive response [31,32]. In the present study, oligonol intake attenuated heat-induced increases in these cytokines. However, there was some discrepancy between levels of inflammation and leukocytosis or lymphocytosis after heat loading. In this regard, future research should consider other factors to explain this discrepancy.

In the present study, a relatively small increase in body temperature (<38 °C) through passive heating was sufficient to stimulate immune system activity. Greater leukocytosis and lymphocytosis at relatively low levels of thermal stress (low tympanic temperature) were observed in the oligonol trial in comparison to those in the placebo trial. This suggests that oligonol may enhance sensitivity to heat-induced immune activation. In general, temperature plays a crucial role in the proliferation of leukocytes and lymphocytes, but there is some controversy about this fact. Rhind *et al.* [33] investigated the role of hyperthermia in the differential leukocytosis of exercise and reported that the elevation of core temperature during exercise is an important mediator of SNS activation (epinephrine, norepinephrine, and cortisol) but that it is not likely to be the sole stimulus of leukocyte redistribution. In addition, they reported no direct relationship between core temperature and lymphocyte subset mobilization [33]. The present study’s results provide support for no direct relationship between body temperature and lymphocyte distribution.

The neuroendocrine system affects the immune system, including lymphocyte activity, proliferation, and traffic, through the neuroendocrine humoral outflow via the pituitary and through direct neuronal effects via the sympathetic, parasympathetic (cholinergic), and peptidergic/sensory innervation of peripheral tissue [34]. It is well known that an antioxidant improves the ratio of sympathetic to parasympathetic tone through anti-inflammatory activity [35,36,37]. An increase in parasympathetic activity and a decrease in sympathetic activity led to significant increases in WBC counts and NK-cell cytotoxicity, suggesting an improved immune status [38]. This suggests that oligonol, an antioxidant, may enhance the parasympathetic nervous system and lymphocyte proliferation.

The anti-inflammatory and antioxidant activity of oligonol may enhance immunity. Meydani *et al.* [2] demonstrated that long-term supplementation with vitamin E enhances clinically relevant *in vivo* indices of the T-cell-mediated function in healthy elderly subjects, and Wintergerst *et al.* [3] suggested that supplementation with a combination of selected micronutrients (including antioxidant vitamins) supports the body’s natural defense system by enhancing all three levels of immunity, namely the epithelial barrier, cellular immunity, and antibody production. Immune cells produce reactive oxygen species (ROS) necessary for microbiocidal activity and are sensitive to external ROS because of their high polyunsaturated fatty acid content [1]. Therefore, the sufficient intake of vitamins and antioxidants may be essential for the efficient functioning of the immune system [1,2,3].

Recent studies have started to identify natural bioactive substances as alternatives to synthetic drugs to mitigate their unwanted side effects [39,40,41]. These natural products may be considered potential candidates for the bioactivity of antipyretic, analgesic, and anti-inflammatory agents against fever, algesia, and inflammation, which have been associated with several pathological conditions [39,40,41]. Consistent with the findings of these studies, the present study’s results provide support for the immune potential of oligonol and raises its potential use with hyperthermia as an adjunct to chemotherapy in malignant diseases [23].

Future Studies Using a Larger Cohort and also Assessing Cellular Functional Responses will Consolidate in Promoting Our Present Findings.

## 5. Conclusions

The results suggest oligonol as a potential immune support supplement.

## References

[1] Brambilla D., Mancuso C., Scuderi M.R., Bosco P., Cantarella G., Lempereur L., di Benedetto G., Pezzino S., Bernardini R. (2008). The role of antioxidant supplement in immune system, neoplastic, and neurodegenerative disorders: A point of view for an assessment of the risk/benefit profile. Nutr. J..

[2] Meydani S.N., Meydani M., Blumberg J.B., Leka L.S., Siber G., Loszewski R., Thompson C., Pedrosa M.C., Diamond R.D., Stollar B.D. (1997). Vitamin E supplementation and *in vivo* immune response in healthy elderly subjects. A randomized controlled trial. Jama.

[3] Wintergerst E.S., Maggini S., Hornig D.H. (2007). Contribution of selected vitamins and trace elements to immune function. Ann. Nutr. Metab..

[4] Aruoma O.I., Sun B., Fujii H., Neergheen V.S., Bahorun T., Kang K.S., Sung M.K. (2006). Low molecular proanthocyanidin dietary biofactor oligonol: Its modulation of oxidative stress, bioefficacy, neuroprotection, food application and chemoprevention potentials. Biofactors.

[5] Lee J.B., Shin Y.O., Min Y.K., Yang H.M. (2010). The effect of oligonol intake on cortisol and related cytokines in healthy young men. Nutr. Res. Pract..

[6] Shin Y.-O., Lee J.-B., Min Y.-K., Yang H.-M. (2011). Effect of oligonol intake on cortisol and cytokines, and body temperature after leg immersion into hot water. Food Sci. Biotechnol..

[7] Noh J.S., Park C.H., Yokozawa T. (2011). Treatment with oligonol, a low-molecular polyphenol derived from lychee fruit, attenuates diabetes-induced hepatic damage through regulation of oxidative stress and lipid metabolism. Br. J. Nutr..

[8] Kundu J.K., Chang E.J., Fujii H., Sun B., Surh Y.J. (2008). Oligonol inhibits UVB-induced cox-2 expression in HR-1 hairless mouse skin—AP-1 and C/EBP as potential upstream targets. Photochem. Photobiol..

[9] Severs Y., Brenner I., Shek P.N., Shephard R.J. (1996). Effects of heat and intermittent exercise on leukocyte and sub-population cell counts. Eur. J. Appl. Physiol. Occup. Physiol..

[10] Conti B., Tabarean I., Andrei C., Bartfai T. (2004). Cytokines and fever. Front. Biosci..

[11] Fujii H., Sun B., Nishioka H., Hirose A., Aruoma O.I. (2007). Evaluation of the safety and toxicity of the oligomerized polyphenol oligonol. Food Chem. Toxicol..

[12] Sobieska M., Stratz T., Samborski W., Hrycaj P., Mennet P., Müller W. (1993). Interleukin-6 (IL-6) after Whole Body Cryotherapy and Local Hot Mud Pack Treatment. Eur. J. Phys. Med. Rehabil..

[13] Olszewski W.L., Grzelak I., Ziolkowska A., Engeset A. (1989). Effect of local hyperthermia on lymph immune cells and lymphokines of normal human skin. J. Surg. Oncol..

[14] Metz J.R., Huising M.O., Leon K., Verburg-van Kemenade B.M., Flik G. (2006). Central and peripheral interleukin-1 beta and interleukin-1 receptor I expression and their role in the acute stress response of common carp, cyprinus carpio l. J. Endocrinol..

[15] O’Connor K.A., Johnson J.D., Hansen M.K., Wieseler Frank J.L., Maksimova E., Watkins L.R., Maier S.F. (2003). Peripheral and central proinflammatory cytokine response to a severe acute stressor. Brain Res..

[16] Dinarello C.A. (1991). Interleukin-1 and interleukin-1 antagonism. Blood.

[17] Li S., Wang Y., Matsumura K., Ballou L.R., Morham S.G., Blatteis C.M. (1999). The febrile response to lipopolysaccharide is blocked in cyclooxygenase-2^−/−^, but not in cyclooxygenase-1^−/−^ mice. Brain Res..

[18] Shin Y.O., Lee J.B., Song Y.J., Min Y.K., Yang H.M. (2013). Oligonol supplementation attenuates body temperature and the circulating levels of prostaglandin E2 and cyclooxygenase-2 after heat stress in humans. J. Med. Food.

[19] Lange U., Muller-Ladner U., Schmidt K.L. (2006). Balneotherapy in rheumatic diseases—An overview of novel and known aspects. Rheumatol. Int..

[20] Bouchama A., Al Hussein K., Adra C., Rezeig M., Al Shail E., Al Sedairy S. (1992). Distribution of peripheral blood leukocytes in acute heatstroke. J. Appl. Physiol..

[21] Kappel M., Kharazmi A., Nielsen H., Gyhrs A., Pedersen B.K. (1994). Modulation of the counts and functions of neutrophils and monocytes under *in vivo* hyperthermia conditions. Int. J. Hyperth..

[22] Pedersen B.K., Kappel M., Klokker M., Nielsen H.B., Secher N.H.  (1994). The immune system during exposure to extreme physiologic conditions. Int. J. Sports Med..

[23] Atanackovic D., Nierhaus A., Neumeier M., Hossfeld D.K., Hegewisch-Becker S. (2002). 41.8 Degrees C whole body hyperthermia as an adjunct to chemotherapy induces prolonged T cell activation in patients with various malignant diseases. Cancer Immunol. Immunother..

[24] Ostapenko V.V., Tanaka H., Miyano M., Nishide T., Ueda H., Nishide I., Tanaka Y., Mune M., Yukawa S. (2005). Immune-related effects of local hyperthermia in patients with primary liver cancer. Hepato Gastroenterol..

[25] Tomiyama-Miyaji C., Watanabe M., Ohishi T., Kanda Y., Kainuma E., Bakir H.Y., Shen J., Ren H., Inoue M., Tajima K. (2007). Modulation of the endocrine and immune systems by well-controlled hyperthermia equipment. Biomed. Res..

[26] Huang Y.H., Haegerstrand A., Frostegard J. (1996). Effects of *in vitro* hyperthermia on proliferative responses and lymphocyte activity. Clin. Exp. Immunol..

[27] Downing J.F., Taylor M.W. (1987). The effect of *in vivo* hyperthermia on selected lymphokines in man. Lymphokine Res..

[28] Shen R.N., Hornback N.B., Shidnia H., Shupe R.E., Brahmi Z. (1987). Whole-body hyperthermia decreases lung metastases in lung tumor-bearing mice, possibly via a mechanism involving natural killer cells. J. Clin. Immunol..

[29] Jiang Q., Detolla L., Singh I.S., Gatdula L., Fitzgerald B., van Rooijen N., Cross A.S., Hasday J.D. (1999). Exposure to febrile temperature upregulates expression of pyrogenic cytokines in endotoxin-challenged mice. Am. J. Physiol..

[30] Kluger M.J., Rudolph K., Soszynski D., Conn C.A., Leon L.R., Kozak W., Wallen E.S., Moseley P.L. (1997). Effect of heat stress on LPS-induced fever and tumor necrosis factor. Am. J. Physiol..

[31] Tilg H., Dinarello C.A., Mier J.W. (1997). IL-6 and APPs: Anti-inflammatory and immunosuppressive mediators. Immunol. Today.

[32] Hegde S., Pahne J., Smola-Hess S. (2004). Novel immunosuppressive properties of interleukin-6 in dendritic cells: Inhibition of NF-kappab binding activity and CCR7 expression. FASEB J..

[33] Rhind S.G., Gannon G.A., Shek P.N., Brenner I.K., Severs Y., Zamecnik J., Buguet A., Natale V.M., Shephard R.J., Radomski M.W. (1999). Contribution of exertional hyperthermia to sympathoadrenal-mediated lymphocyte subset redistribution. J. Appl. Physiol..

[34] Elenkov I.J. (2008). Neurohormonal-cytokine interactions: Implications for inflammation, common human diseases and well-being. Neurochem. Int..

[35] Monteiro M.M., Franca-Silva M.S., Alves N.F., Porpino S.K., Braga V.A. (2012). Quercetin improves baroreflex sensitivity in spontaneously hypertensive rats. Molecules.

[36] Giusti M.F., Sato M.A., Cardoso L.M., Braga V.A., Colombari E. (2011). Central antioxidant therapy inhibits parasympathetic baroreflex control in conscious rats. Neurosci. Lett..

[37] Manzella D., Barbieri M., Ragno E., Paolisso G. (2001). Chronic administration of pharmacologic doses of vitamin E improves the cardiac autonomic nervous system in patients with type 2 diabetes. Am. J. Clin. Nutr..

[38] Saeki Y., Nagai N., Hishinuma M. (2007). Effects of footbathing on autonomic nerve and immune function. Complement. Ther. Clin. Pract..

[39] Dos Santos M.D., Almeida M.C., Lopes N.P., de Souza G.E. (2006). Evaluation of the anti-inflammatory, analgesic and antipyretic activities of the natural polyphenol chlorogenic acid. Biol. Pharm. Bull..

[40] Muhammad N., Saeed M., Khan H. (2012). Antipyretic, analgesic and anti-inflammatory activity of viola betonicifolia whole plant. BMC Complement. Altern. Med..

[41] Shilpi J.A., Islam M.E., Billah M., Islam K.M., Sabrin F., Uddin S.J., Nahar L., Sarker S.D. (2012). Antinociceptive, anti-inflammatory, and antipyretic activity of mangrove plants: A mini review. Adv. Pharmacol. Sci..

